# ReqTestAgent: execution-grounded language-model agents for acceptance testing under requirement ambiguity

**DOI:** 10.3389/frai.2026.1960601

**Published:** 2026-10-19

**Authors:** Yunjiao Shi, Rui Song

**Affiliations:** 1 Faculty of Engineering, The University of Hong Kong, Hong Kong, Hong Kong SAR, China; 2 Department of Earth Sciences, University of Cambridge, Cambridge, United Kingdom

**Keywords:** acceptance testing, automated test generation, language-model agents, large language models, mutation testing, requirement ambiguity, requirements engineering, retrieval-augmented generation

## Abstract

**Introduction:**

Large language models (LLMs) can translate ambiguous requirements into executable tests whose assertions conflict with the accepted behavior. Evaluating such tests requires distinguishing behavioral compatibility from fault-detection strength.

**Methods:**

We present ReqTestAgent, an execution-grounded workflow that connects requirement interpretation, testing-knowledge retrieval, test generation, and feedback from canonical execution and mutation testing. We evaluate 100 requirement instances formed from 25 Orchid/BigCodeBench tasks, each with lexical, semantic, syntactic, and vagueness variants, using a reference implementation available to the evaluator. Comparisons include three generation baselines, their verifier-enhanced counterparts, and component ablations.

**Results:**

The generation baselines fail the canonical implementation on 51%–68% of instances, whereas all verifier-enhanced methods achieve 100% canonical pass. ReqTestAgent achieves 92% qualified suites and a macro-averaged effective mutation score of 0.678, compared with 0.655–0.670 for the verifier-enhanced baselines. Removing verification reduces its effective score to 0.216. Across instance–mutant evaluations, ReqTestAgent kills 492 of 756 mutants, a pooled rate of 0.651; this rate weights instances by their mutant counts. A comparative execution trace shows how explicit domain assertions detect faults missed by the verifier-enhanced baselines.

**Discussion:**

Execution feedback provides the principal validity improvement in this controlled setting. The workflow links interpretation records to executable checks, while the evaluation establishes canonical compatibility as a prerequisite for interpreting mutation-based test strength.

## 1 Introduction

An acceptance test turns a statement of required behavior into a decision about whether software is acceptable. When that statement admits several interpretations, an LLM can produce precise assertions without establishing which interpretation they represent. A test may execute successfully yet reject the accepted implementation, or it may accept that implementation through assertions too weak to detect a behavioral fault. These outcomes make requirement ambiguity an oracle-design problem as well as a language-understanding problem. Research on nocuous ambiguity, requirements quality, and testability identifies the textual conditions that create this risk (Chantree et al., 2006; Zhao et al., 2021; Zakeri-Nasrabadi and Parsa, 2024); the challenge for test generation is to connect a chosen interpretation to evidence about executable behavior.

Requirements-based test generation has established methods for deriving system and acceptance tests from use cases and natural-language conditions (Wang et al., 2015, 2022; Fischbach et al., 2023). LLM-based approaches expand the range of descriptions that can be processed, and retrieval and iterative feedback provide mechanisms for improving generated tests (Schäfer et al., 2024; Yuan et al., 2024; Wang et al., 2025). For ambiguous inputs, their evaluation must resolve two linked questions: whether the generated oracle is compatible with an accepted behavioral reference, and whether a compatible oracle detects meaningful deviations. Counting executable tests or measuring fault detection without a validity gate can obscure this distinction (Barr et al., 2015; Inozemtseva and Holmes, 2014).

We study these questions in a setting where an accepted implementation is available for evaluation, as in regression testing or the validation of an existing software component. ReqTestAgent makes the path from requirement interpretation to executable checking inspectable. An ambiguity critic and a repair stage articulate the interpretation used for generation; retrieved testing knowledge supplies candidate test strategies; canonical execution guides test repair; and mutation feedback directs additional checks toward undetected behavioral changes. The workflow builds on established retrieval and feedback mechanisms (Lewis et al., 2020; Yao et al., 2023; Madaan et al., 2023) and retains the intermediate artifacts that connect their decisions to the resulting assertions.

The empirical study uses 25 Orchid/BigCodeBench tasks, each represented by four source-provided ambiguity variants. We compare ReqTestAgent with direct prompting, structured test-design prompting, vanilla RAG, and verifier-enhanced versions of all three. This design locates the contribution of execution feedback within a broader workflow. The results show a substantial canonical-validity gap in the generation-only methods and complete canonical validity among the verifier-enhanced methods. Ablations and task-level paired comparisons then establish which differences in mutation strength the available evidence supports.

The study makes three contributions. First, it operationalizes the evaluation of tests generated from ambiguous requirements through a canonical-validity gate followed by mutation-based assessment. Second, it implements a traceable execution-grounded workflow, preserving requirement interpretations, testing guidance, generated assertions, and feedback-driven revisions. Third, it provides a controlled empirical account of this workflow through strong verifier baselines, component ablations, a second model endpoint, and comparative execution traces. These contributions address three research questions: *RQ1*, how generation and verification affect validity and fault detection; *RQ2*, how the component configurations and their traces explain observed outcomes; and *RQ3*, how these outcomes vary across ambiguity classes and model endpoints, and what model-request time they require.

## 2 Related work

### 2.1 Requirement ambiguity and acceptance-test generation

Ambiguity in natural-language requirements becomes problematic when readers can make different implementation or validation decisions from the same statement. Chantree et al. (2006) formalized this concern through the notion of nocuous ambiguity in requirements. NLP has since been widely applied across requirements-engineering tasks, including analysis of requirements quality and ambiguity-related phenomena (Zhao et al., 2021). Requirements-smell and defect-detection studies provide a related quality-assurance view: textual patterns can expose requirements that are vague, unverifiable, or otherwise risky for downstream engineering work (Femmer et al., 2017; Ferrari et al., 2018). More recent work on natural-language requirement testability connects testability problems with requirement smells, including ambiguous, context-sensitive, and vague language (Zakeri-Nasrabadi and Parsa, 2024). LLM-based assistance has also been explored for improving requirements completeness (Luitel et al., 2024). These findings explain why ambiguous or weakly testable requirements create unstable inputs for downstream test generation.

Requirements-based test generation turns textual requirements into executable checks or test cases. Earlier work generated system tests from use case specifications, and later work extended this line to acceptance-test generation with NLP-based processing of use cases (Wang et al., 2015, 2022). Fischbach et al. (2023) studied automatic creation of acceptance tests by extracting conditionals from requirements. Recent work has also examined retrieval-augmented generation for software requirement-based test case generation (Wang et al., 2025). ReqTestAgent studies how explicit interpretation and execution feedback connect ambiguous requirement inputs to canonically valid, mutation-sensitive tests. Its evaluation retains verifier-enhanced baselines to measure the effect of canonical execution directly.

### 2.2 LLM-based test generation

LLMs have recently been evaluated for automated unit-test generation in software engineering. Schäfer et al. (2024) empirically evaluated LLMs for automated unit-test generation, and Yuan et al. (2024) evaluated and improved ChatGPT for unit-test generation. Broader surveys and empirical studies show that LLM-based software testing and LLM-for-software-engineering research now cover test generation, evaluation, and open methodological problems (Fan et al., 2023; Wang et al., 2024; Yang L. et al., 2024b). LLMs have also been combined with automated test-generation techniques, as in CodaMosa, and with mutation testing for test generation (Lemieux et al., 2023; Dakhel et al., 2024). Acceptance-test generation inherits an additional requirement-level risk because executable assertions can reflect an unintended reading of the requirement. This distinction motivates separate reporting of canonical validity and mutation-based strength.

### 2.3 Retrieval-augmented and agentic LLM workflows

Retrieval-augmented generation conditions a language model on retrieved external knowledge rather than relying only on parametric memory (Lewis et al., 2020). Recent RAG surveys characterize this as a general pattern for coupling retrieval with generation in LLM systems (Fan et al., 2024). Agentic LLM workflows further interleave reasoning with actions or feedback, as in ReAct-style reasoning/action loops (Yao et al., 2023), tool-use methods (Schick et al., 2023), and iterative verbal or self-feedback methods (Shinn et al., 2023; Madaan et al., 2023). Related software-engineering work has studied agent-computer interfaces and collaboration between intelligent agents and LLMs for coding tasks (Yang J. et al., 2024a; Bai et al., 2025). These lines of work inform the retrieval and refinement stages of ReqTestAgent, where testing knowledge guides generation and executable outcomes guide subsequent edits.

### 2.4 Mutation testing

Mutation testing evaluates a test suite by checking whether it detects small program changes, and it has a long history as a testing technique (Jia and Harman, 2011; Papadakis et al., 2019). Mutants have also been studied as substitutes for real faults, with known limitations (Just et al., 2014). The choice is consistent with evidence that simple coverage measures should not be treated as strong proxies for test-suite effectiveness (Inozemtseva and Holmes, 2014). In this study, canonical pass acts as a validity gate and mutation score estimates fault-detection strength; invalid suites receive an effective score of zero.

## 3 Materials and methods

### 3.1 Task formulation

Given an ambiguous requirement and its public task context, the objective is to generate an executable test suite that accepts a designated reference implementation and detects behavioral perturbations of it. The reference implementation supplies the canonical behavior for this evaluation. Its availability is a condition of the execution-based workflow. Canonical compatibility and mutation sensitivity are assessed separately so that failures caused by an incorrect oracle do not count as successful fault detection.

All methods use a common public task view. Initial prompts contain the ambiguous requirement, ambiguity class, function signature and entry point, library metadata, and structured documentation fields for parameters, returns, exceptions, requirements, examples, and notes. The source ambiguity explanation additionally informs the retrieval query in retrieval-based methods. The complete prompt, canonical solution, and original tests are reserved for the evaluator and excluded as initial input fields. This setting evaluates generation with annotated requirement context and public examples.

### 3.2 ReqTestAgent architecture

ReqTestAgent coordinates an ambiguity critic, a requirement repair stage, a testing-knowledge retriever, a test generator, and execution-based refinement (Figure 1). The critic identifies unclear terms, missing boundaries, and oracle choices. The repair stage turns these observations into an explicit testable interpretation, which is passed to the generator and retained in the trace. Thus, repair has an operational role in generation and a documentary role in recording assumptions. The retriever supplies compact guidance on boundary-value analysis, exception contracts, oracle design, metamorphic checks, and domain-specific testing. The generator combines this guidance with the public task context and interpretation to produce pytest- or unittest-compatible tests.

**Figure 1:**
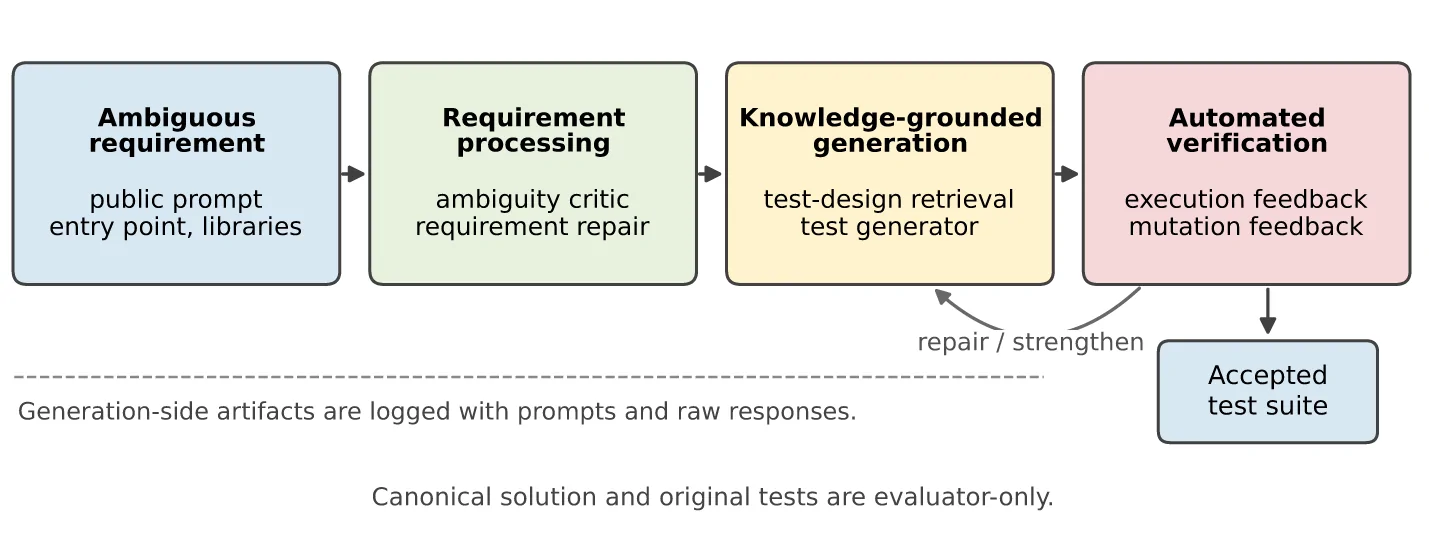
Overview of ReqTestAgent. The generation side receives the public task context, repaired interpretation, and retrieved testing knowledge; execution diagnostics guide refinement. The complete canonical implementation and original tests are evaluator-side inputs and are excluded from initial generation prompts.

The agent roles organize the stages and their intermediate outputs. Their empirical contribution is assessed through the component configurations in Section 3.6.

### 3.3 Verifier loop and mutation feedback

The verifier executes a generated suite against the canonical implementation and returns failure diagnostics for test repair. If the repaired suite still fails, the evaluator checks individual tests and retains passing tests when a nonempty subset is available. If no tests survive, a further repair is requested and checked, with another filtering pass if needed. The final suite is then evaluated for both canonical validity and test strength. Recording the number of tests, assertions, and detected mutants makes the effect of filtering visible in the quality assessment.

For a canonical-valid suite below the configured mutation target, mutation feedback reports the score and counts of surviving mutation categories and requests additional or revised checks. A candidate replaces the current suite only if it remains canonical-valid and increases the main-pool mutation score. Held-out mutants are excluded from feedback and candidate selection. In the compared implementations, ReqTestAgent and its w/o Repair and w/o RAG ablations use this stage; the three verifier-enhanced generation baselines use canonical feedback and filtering. The comparison therefore evaluates the complete configurations, with component effects examined through the ablations.

### 3.4 Traceability and leakage control

Each run stores the request, model response, parsed test code, provider-reported model identifier, generation parameters, timestamp, token metadata, execution results, and mutant outcomes. The trace also retains ambiguity diagnoses, repaired interpretations, retrieved knowledge files, and intermediate suites. These records allow an assertion to be inspected alongside the interpretation and feedback that preceded it. Verification returns execution diagnostics, which can include traceback excerpts; mutation feedback returns aggregate outcomes without mutant source. The complete reference implementation and original test suite remain evaluator-side inputs.

### 3.5 Evaluation set and experimental setup

The evaluation set is derived from the cached Orchid-BCB release, which contains 164 BigCodeBench-based tasks. BigCodeBench supplies function-level tasks with diverse library calls and detailed instructions (Zhuo et al., 2025); Orchid supplies ambiguity variants for studying interpretation under requirement ambiguity (Yang et al., 2026). We shuffled the source tasks with seed 20260618 and selected the first 25 eligible tasks. Eligibility required available dependencies, compatibility with the execution restrictions, and passing original tests on the canonical implementation. During selection, six encountered tasks were excluded because of missing libraries, restricted process or network operations, or a failing original test. The supplementary selection record identifies the tasks and reasons.

For each selected task, we retained Orchid's existing lexical, semantic, syntactic, and vagueness prompts and their corresponding explanations. No additional rewriting of those prompts was used to construct the evaluation set. Expanding each base task into its four source variants yields 100 instances, with 25 instances per class. Lexical ambiguity concerns the meaning or referent of an expression; semantic ambiguity concerns the behavior implied by a statement; syntactic ambiguity concerns how grammatical structure connects conditions or actions; and vagueness leaves a behavioral boundary or criterion insufficiently specified. Table 1 illustrates these categories using the retained prompts. The labels follow the source dataset, and the four variants share the same canonical implementation and original tests.

**Table 1:** Source-defined ambiguity classes and examples retained from Orchid-BCB.

Class	Interpretation issue	Example and resulting test decision
Lexical	Meaning or referent of an expression	P011: “it is an object” leaves the sales-value type unclear; tests must choose which returned type to assert.
Semantic	Behavior implied by a statement	P005: “unique character occurrence across all strings” permits character multiplicity or per-string incidence; the count oracle differs.
Syntactic	Attachment of conditions or modifiers	P009: “the sum of the integers in T1 between 0 and RANGE” leaves the range modifier's attachment unclear; the range may constrain the generated integers or the input sum.
Vagueness	Insufficiently specified criterion	P005: “specified ASCII characters” does not identify the character set; tests must determine the accepted alphabet.

Each class contains 25 instances, one for each selected base task. The requirement excerpts are reproduced from Orchid-BCB (Yang et al., 2026; https://huggingface.co/datasets/YDDYES/Orchid), licensed under the Apache License 2.0.

The main evaluation covers all 100 instances. The secondary-model evaluation uses the first 10 selected base tasks and all four variants, giving a fixed 40-instance subset. The main evaluation used an OpenAI-compatible API reporting gpt-5.4-mini; the secondary evaluation used a separate compatible endpoint reporting deepseek-v4-flash. All calls used temperature 0.0 and were made in June 2026. The artifact records the returned model identifiers, parameters, timestamps, token counts, and responses.

The original evaluation environment used Python 3.10.5 (Python Software Foundation, Beaverton, OR, USA) with pandas 2.1.0, NumPy 1.24.3, Matplotlib 3.8.0, SciPy 1.11.4, scikit-learn 1.3.2, seaborn 0.13.2, and pytest 9.1.0 on Windows 10. Its environment snapshot is included in the supplementary artifact, together with instructions for analyzing cached results and replaying saved tests without accessing a model endpoint.

### 3.6 Compared methods

The comparison includes direct prompting, structured test-design prompting, vanilla RAG, verifier-enhanced versions of these three methods, ReqTestAgent, and three ablations. Structured prompting, identified as ghl in the artifact, asks the model to select relevant test-design techniques and cover normal, boundary, negative, and oracle-specific cases. Vanilla RAG adds retrieved testing guidance. Their verifier-enhanced counterparts use the canonical repair and filtering procedure described above. All methods use the same public input fields and evaluator.

The w/o Repair configuration retains the critic, retrieval, generation, and execution stages while omitting explicit requirement repair. The w/o RAG configuration omits retrieval. The w/o Verifier configuration retains the interpretation and generation stages but omits execution-based refinement, including mutation feedback. These configurations distinguish the execution contribution from the changes associated with interpretation and retrieval.

### 3.7 Evaluation metrics and analysis

We report executability, canonical pass, qualified-suite rate, mutation-based strength, and model-request time. Executability requires the absence of syntax and import failures. A canonical-valid suite passes the reference implementation; the invalid-suite rate is its complement. A qualified suite must be canonical-valid, contain more than one test and at least one assertion, and attain a mutation score of at least 0.3 when mutants are available. All selected tasks have main-pool mutants.

Let *v*_*i*_ indicate canonical validity for instance *i*, and let *k*_*i*_ and *m*_*i*_ denote the numbers of killed and evaluated main-pool mutants. The effective score and its macro-average are:


$$e_{i}=\{\begin{array}{cc} \frac{k_{i}}{m_{i}} & v_{i}=1,\\ 0 & v_{i}=0, \end{array}\;\bar{e}=\frac{1}{N}\sum_{i=1}^{N}e_{i}.$$ (1)

Each instance has equal weight in $\bar{e}$. The pooled kill rate, $\underset{i}{\sum }k_{i}/\underset{i}{\sum }m_{i}$, instead weights evaluated instances by their numbers of mutants. A valid-suite mean averages *k*_*i*_/*m*_*i*_ over canonical-valid suites. A held-out mean averages held-out scores over canonical-valid instances with at least one held-out mutant; we report that denominator explicitly. These measures separate compatibility from the fault-detection evidence obtained after compatibility has been established (Barr et al., 2015; Jia and Harman, 2011; Just et al., 2014; Papadakis et al., 2019).

Inferential comparisons use 25 paired task-level observations, each averaging the four effective scores for one base task. We report mean paired differences and 95% bootstrap confidence intervals from 10,000 resamples of base tasks (seed 0), together with two-sided Wilcoxon signed-rank tests and Holm correction across nine comparisons with ReqTestAgent. Descriptive rates use the 100 instances. This treatment preserves the dependence among ambiguity variants of the same task.

The mutant generator applies AST-level changes to comparisons, boolean conditions, numeric and string constants, binary operators, and return values, discarding duplicate and uncompilable candidates. The main pool contains at most 10 mutants per base task. Later candidates form the held-out pool and are excluded from feedback and selection. Auditing all 25 tasks yielded 281 candidate sites and 189 main-pool mutants, with at least one mutant per task. Original tests passed all canonical implementations and killed 104 of 189 mutants (pooled rate 0.550; macro-average 0.602). Each task's mutants are reused for its four requirement variants, so the 100-instance comparison comprises 756 instance–mutant evaluations.

Recorded time is the sum of elapsed model-request times for an instance, including refinement requests. It excludes local retrieval, canonical execution, and mutant execution, and therefore does not measure end-to-end runtime. Token usage is reported from provider metadata. Gateway-specific pricing was unavailable, so the recorded cost fields are not interpreted as monetary estimates.

## 4 Results

### 4.1 Main comparison

Table 2 reports the 100-instance comparison. All methods achieve an executable rate of 1.000, but direct prompting, structured test-design prompting, and vanilla RAG fail the canonical implementation on 51%, 68%, and 66% of instances, respectively. Their effective mutation scores are 0.361, 0.250, and 0.261. All three verifier-enhanced baselines achieve canonical pass of 1.000, with effective scores of 0.655–0.670. ReqTestAgent also achieves canonical pass of 1.000, with a qualified-suite rate of 0.920 and an effective score of 0.678 (Figure 2).

**Table 2:** Main results on the 100-instance controlled evaluation.

Method	**Canon**.	**Invalid**	Qualified	**Eff. Mut**.	**Model time (s)**
Direct	0.490	0.510	0.470	0.361	9.07
Structured prompt	0.320	0.680	0.310	0.250	8.63
Vanilla RAG	0.340	0.660	0.340	0.261	8.97
Direct + verifier	1.000	0.000	0.890	0.655	14.05
Structured + verifier	1.000	0.000	0.900	0.668	15.20
Vanilla RAG + verifier	1.000	0.000	0.940	0.670	15.18
ReqTestAgent	1.000	0.000	0.920	0.678	64.46

Effective mutation is the macro-average defined in Equation 1; invalid suites receive zero. Model time sums all recorded model requests, including rejected candidates.

**Figure 2:**
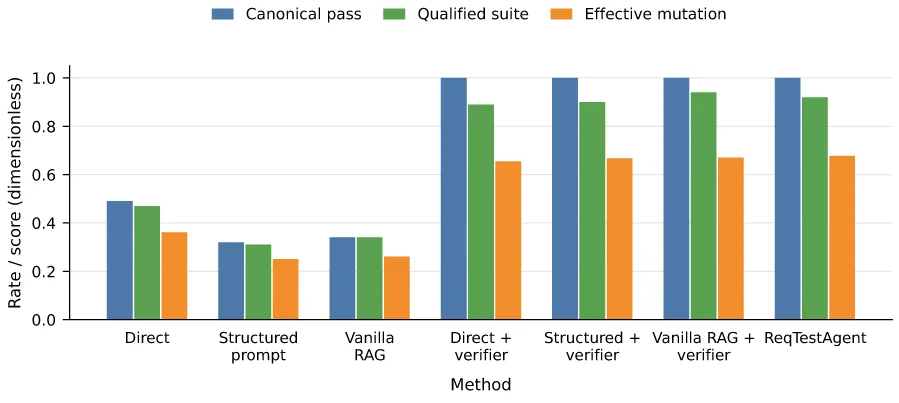
Validity and test-strength results for the main evaluation. The largest gap separates non-verifier methods from verifier-based methods; ReqTestAgent retains the validity gain while recording requirement-level interpretation and retrieval traces.

For ReqTestAgent, 0.678 is the equal-weight mean of the 100 instance-level scores in Equation 1. The corresponding pooled count is 492 kills across 756 instance–mutant evaluations, giving 492/756 = 0.651. Instances contain between 1 and 10 mutants, so equal weighting of instances and weighting by mutant count yield different values. The pooled count comprises repeated evaluation of the 189 base-task mutants across four requirement variants.

Figure 3 characterizes suites that fail the qualification criteria. Direct prompting has 37 canonical assertion failures and 14 canonical runtime failures; structured prompting has 53 and 15, and vanilla RAG has 55 and 11. ReqTestAgent's eight unqualified suites all pass the canonical implementation: seven fall below the mutation threshold, and one is classified as trivial. These results identify two distinct outcomes for generated tests: compatibility with the reference and sufficient assertion strength within the mutation pool.

**Figure 3:**
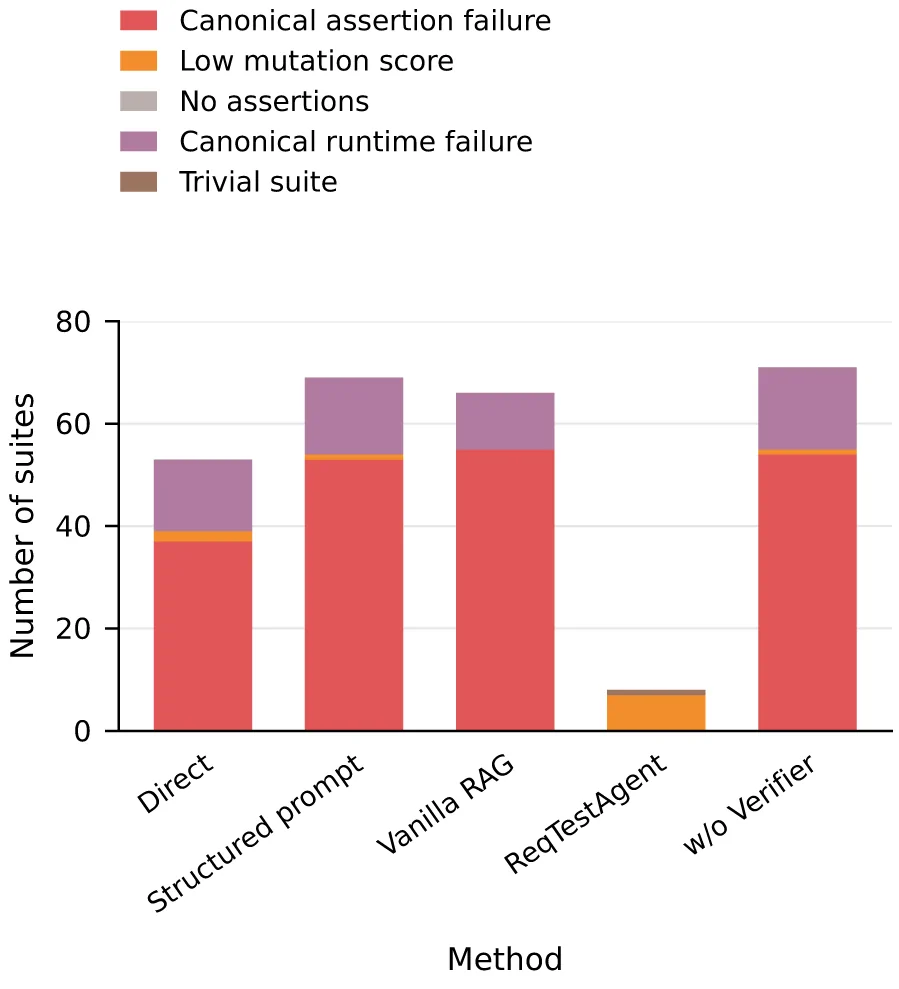
Failure modes among weak or invalid suites. Non-verifier methods mainly fail through assertion or runtime failures on the canonical implementation. ReqTestAgent's eight unqualified suites pass the canonical implementation: seven fall below the mutation threshold and one contains only a single test.

Held-out scores use the covered-instance denominator defined in Section 3.7. Each verifier-enhanced method has held-out mutants on the same 40 of 100 instances, spanning 10 base tasks. The corresponding mean scores are 0.878 for direct + verifier, 0.871 for structured + verifier, 0.890 for vanilla RAG + verifier, and 0.874 for ReqTestAgent. The generation-only methods have 9–12 canonical-valid instances with held-out mutants, so their conditional means describe different subsets. Coverage and conditional means are reported separately in the Supplementary results.

### 4.2 Ablation and statistical analysis

Removing execution-based verification reduces canonical pass from 1.000 to 0.300, qualified-suite rate from 0.920 to 0.290, and effective mutation score from 0.678 to 0.216 (Table 3; Figure 4). The w/o Repair configuration achieves an effective score of 0.695, and w/o RAG achieves 0.664. The full-minus-ablation differences are −0.017 (95% task-bootstrap CI [−0.050, 0.014]) and 0.014 [−0.011, 0.038], respectively. These comparisons do not establish an average mutation-score gain from retaining repair or retrieval in this evaluation.

**Table 3:** Ablation results on the 100-instance controlled evaluation.

Method	**Canon**.	**Qualified**	**Eff. Mut**.	**Model time (s)**
ReqTestAgent	1.000	0.920	0.678	64.46
w/o Repair	1.000	0.920	0.695	55.17
w/o RAG	1.000	0.890	0.664	68.48
w/o Verifier	0.300	0.290	0.216	27.24

Model time includes every saved request; mutation feedback is omitted in w/o Verifier.

**Figure 4:**
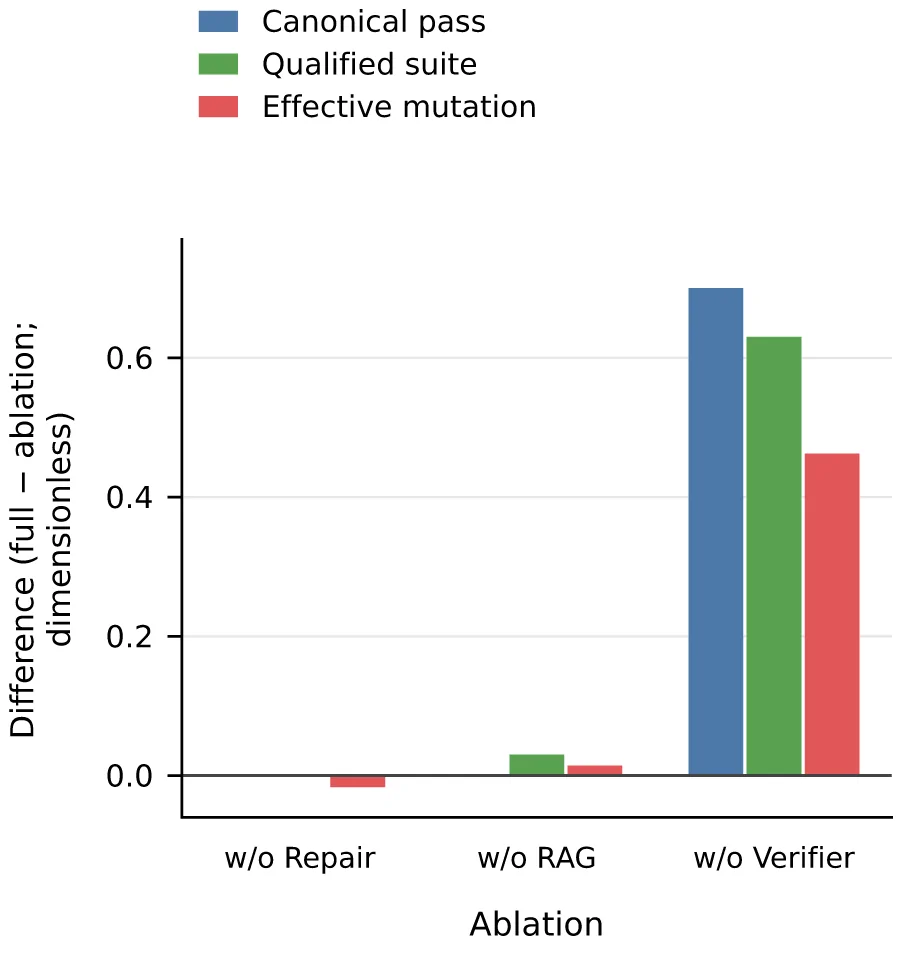
Ablation effects relative to ReqTestAgent. Values are full-configuration scores minus ablation scores. Removing verification changes canonical validity and mutation strength most; the repair and RAG score differences have task-bootstrap intervals spanning zero.

Task-level paired comparisons show effective-score differences of 0.317, 0.428, 0.417, and 0.462 between ReqTestAgent and direct prompting, structured prompting, vanilla RAG, and w/o Verifier, respectively (Holm-adjusted *p* < 0.001 in each case). The difference against direct + verifier is 0.023 (95% CI [−0.004, 0.056]; unadjusted *p* = 0.110, Holm-adjusted *p* = 0.549). The differences against structured + verifier and vanilla RAG + verifier are 0.011 [−0.023, 0.047] and 0.008 [−0.018, 0.039], with adjusted *p* = 1.000 in both cases. The available task-level evidence supports the validity contribution of execution feedback without establishing a population-level mutation-score advantage over the verifier-enhanced baselines.

Across the four ambiguity classes, ReqTestAgent retains canonical pass of 1.000 and effective mutation scores from 0.666 to 0.691 (Figure 5). Qualified-suite rates range from 0.880 for lexical instances to 0.960 for syntactic instances. On the 10 held-out-covered instances per class, mean held-out scores are 0.871 for lexical, 0.815 for semantic, and 0.905 for both syntactic and vagueness variants. These class-level results describe performance under the four source-defined perturbations.

**Figure 5:**
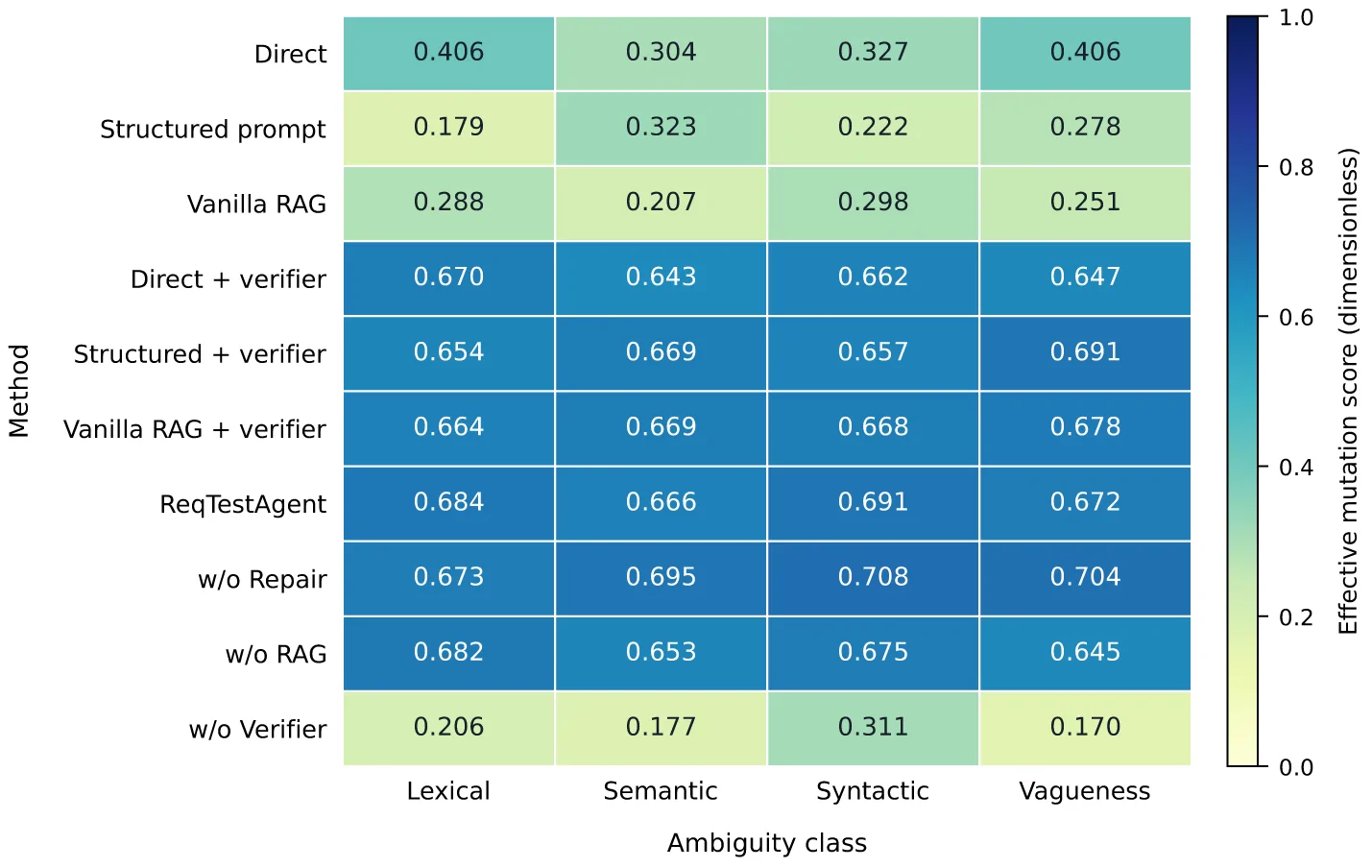
Effective mutation score by ambiguity type. Each cell averages 25 instances. The complete method comparison shows the canonical-validity contribution of execution feedback across all four source-defined classes.

### 4.3 Secondary-model evaluation

The fixed 40-instance evaluation on the second endpoint preserves complete canonical validity for all three verifier-centered methods (Table 4). ReqTestAgent achieves a qualified-suite rate of 1.000 and an effective mutation score of 0.800, compared with 0.900 and 0.720 for direct + verifier and 0.900 and 0.790 for vanilla RAG + verifier. Held-out mutants are available for the same 16 instances, spanning four base tasks, for each method. Their conditional mean held-out scores are 0.831, 0.706, and 0.750, respectively. This descriptive comparison extends the observed canonical-validity pattern to a second provider-reported model and records its associated mutation-strength results.

**Table 4:** Secondary-model evaluation on the fixed 40-instance subset.

Method	**Canon**.	**Qualified**	**Eff. Mut**.	**Held-out**	Model time (s)
Direct + verifier	1.000	0.900	0.720	0.706	60.62
Vanilla RAG + verifier	1.000	0.900	0.790	0.750	59.91
ReqTestAgent	1.000	1.000	0.800	0.831	243.25

Held-out scores are conditional means over the same 16 instances with available held-out mutants. Model time includes every saved request.

### 4.4 Case analysis

Two traces from the existing main evaluation illustrate how the complete workflow detects changes missed by the verifier-enhanced baselines (Table 5). These cases were selected after inspecting the 100-instance results for additional detected faults. Replaying all four saved suites for each case reproduced their canonical passes and both main-pool and held-out mutant outcomes without generating new tests.

**Table 5:** Comparative traces from the main evaluation.

Method	P018-Syntactic	P011-Semantic
Direct + verifier	0.700/0.800	0.600/1.000
Structured + verifier	0.300/0.400	0.600/1.000
Vanilla RAG + verifier	0.400/0.400	0.600/1.000
ReqTestAgent	0.900/0.800	0.800/1.000

Every listed suite passes the canonical implementation. Cells report main-pool/held-out mutation scores. P018 has 10 main and 10 held-out mutants; P011 has 10 main and 1 held-out mutant.

In P018-Syntactic (BigCodeBench/143), the requirement specifies a plot of *y* = 2*x*+1 over *x*∈[−10, 10] with a green marker at (2, 5). ReqTestAgent's suite checks that the minimum plotted *x* is at least −10 and the maximum is at most 10. Direct + verifier instead checks that the plotted data reach at least as far as those endpoints: minimum *x* ≤ −10 and maximum *x*≥10. Both suites pass the canonical implementation, but the latter inequalities accept an expanded domain. Mutants M01 and M02 change the endpoint constants to extend the generated data beyond the specified interval. The full suite detects both, raising its main-pool score to 0.900, compared with 0.700, 0.300, and 0.400 for the direct, structured, and RAG verifier baselines. This example connects the observable requirement constraint to the direction of the assertion and the detected behavioral changes.

In P011-Semantic (BigCodeBench/88), the task generates daily sales from a seeded random process. The public documentation includes an example with sales values [102, 435, 348, 270, 106]. Following canonical repair, the full workflow adds an example-based check during mutation feedback and achieves a main-pool score of 0.800; all three verifier-enhanced baselines achieve 0.600. The added check detects changes to the seed and to the lower bound of the sampled range. The expected values originate in the public example available to every method. This trace demonstrates how refinement can turn an available behavioral example into an additional oracle.

The component ablations achieve 0.900 on the plotting case, while w/o Repair and w/o RAG achieve 0.700 and 0.600 on the sales case. The cases illustrate the observed complete-configuration outcomes; component-level conclusions are determined by the task-level comparisons above.

### 4.5 Model-request time and token usage

ReqTestAgent makes 657 model requests across the 100 main instances. Summing the elapsed time of every saved request, including candidates that are subsequently rejected, gives a mean of 64.46 s per instance, compared with 14.05–15.20 s for the verifier-enhanced baselines. Its 962,737 input tokens and 473,010 output tokens accompany requests for interpretation, generation, and refinement. The mean time comprises 15.34 s for ambiguity diagnosis and requirement repair, 11.04 s for initial test generation, 7.21 s for canonical repair, and 30.87 s for mutation-directed additions, rewrites, and associated repairs. Retrieval is performed locally.

Relative to direct + verifier, the full configuration uses 4.59 times the model-request time for an observed effective-score difference of 0.023. In the secondary evaluation, mean request times are 243.25 s for ReqTestAgent, 60.62 s for direct + verifier, and 59.91 s for vanilla RAG + verifier. These figures use all saved requests; local execution time and monetary charges are outside the reported measurement. The supplementary artifact provides per-request and per-stage totals, including rejected candidates, for reproduction of this accounting.

## 5 Discussion

The results support an execution-grounded account of test generation under requirement ambiguity. Across the three generation baselines, syntax and import checks accept every suite, while canonical execution rejects 51%–68%. Adding the verifier raises canonical pass to 100% for all three baselines as well as ReqTestAgent. The practical contribution of the validity gate is to connect the generated oracle to an accepted behavioral reference before mutation kills are interpreted as evidence of test quality.

Mutation analysis adds a second decision: whether an accepted suite constrains the behavior precisely enough to detect faults. In the comparative plotting case, assertions on the generated data expose an expanded input domain that remains compatible with weaker plot checks. The trace links the requirement's numerical interval, the assertion direction, and the additional detected mutants. This provides a concrete account of a full-workflow advantage on an observed instance. The aggregate comparisons in Section 4.2 determine the broader scope of that advantage.

Requirement interpretation and retrieved guidance contribute inspectable intermediate artifacts. The repair stage supplies the generator with an explicit interpretation, and the trace preserves that interpretation alongside the resulting tests. Retrieved notes document which testing strategies informed generation. These artifacts enable an engineer to examine why a boundary or expected value appears in a suite. Their presence is established by the execution records; the component comparisons quantify their observed relationship to mutation strength.

The resource measurements make the deployment choice explicit. Direct + verifier achieves complete canonical validity, an effective score of 0.655, and 14.05 s of model-request time. ReqTestAgent achieves the same validity, an effective score of 0.678, and 64.46 s. The full workflow allocates additional requests to requirement interpretation and mutation-directed refinement and produces corresponding intermediate records. A verifier-enhanced generator is therefore a supported configuration when canonical-valid tests and short turnaround are the primary requirements. The full workflow is a candidate when the interpretation record and targeted assertion refinement are needed. The present measurements quantify the computational trade-off; they do not establish a reduction in human review effort or monetary cost.

### 5.1 Scope and applicability

The execution-based workflow requires an accepted reference implementation. This condition fits regression testing and validation of an existing component, where the implementation can ground executable acceptance checks. During early requirements engineering, a golden implementation may not yet exist; a stakeholder-approved specification, executable model, or other independent oracle would be needed to provide comparable behavioral evidence. Canonical pass establishes compatibility with the chosen reference and cannot by itself establish agreement among stakeholders. Our evaluation concerns function-level Python tasks. System acceptance additionally involves component integration, external I/O, stateful interactions, and potentially conflicting stakeholder expectations, which require evaluation beyond the present task setting.

### 5.2 Limitations and further evaluation

The controlled dataset reuses source-provided ambiguity variants and annotations from 25 base tasks. Its public context includes structured documentation and examples. The findings therefore apply to generation with this context, while independently authored requirements and richer system interfaces would test transfer to other settings. The secondary endpoint reproduces the canonical-validity pattern on a fixed 10-task subset and supplies a further descriptive comparison of test strength.

Mutation scores describe detection within the specified pools. Surviving mutants may include equivalent or low-value changes, and the 189 base-task mutants are reused across requirement variants (Just et al., 2014; Papadakis et al., 2019). Task-level inference accounts for the shared base tasks. The held-out analysis uses later candidates from the same generator and reports its coverage separately, so its evidence concerns this additional mutation pool.

Hosted model endpoints may change or become unavailable. The archived prompts, responses, generated tests, and evaluator outputs support result reanalysis and test replay independently of continued model access. Fresh generation with a replacement model constitutes a new experimental condition and should be reported separately. Further evaluation can examine whether the recorded interpretation traces reduce engineering effort and which requirement characteristics predict a benefit from additional refinement.

## 6 Conclusion

ReqTestAgent connects requirement interpretation to acceptance-test generation through canonical execution, mutation feedback, and retained intermediate records. On 100 ambiguity-controlled instances from 25 tasks, the evaluation distinguishes executable tests from canonically valid tests and then measures their fault-detection strength. Execution-based verification provides the principal validity improvement: all verifier-enhanced methods accept the reference implementation on every instance, and ReqTestAgent achieves a macro-averaged effective mutation score of 0.678. Comparative traces explain how additional behavioral constraints can detect faults missed by simpler verifier-enhanced configurations.

The resulting contribution is an inspectable workflow and an empirical basis for assessing tests generated from ambiguous requirements when a behavioral reference is available. Canonical execution grounds the oracle, mutation analysis evaluates its discriminatory strength, and the trace connects both outcomes to the requirement interpretation. This sequence provides a basis for extending the approach to richer acceptance-testing settings and for evaluating the engineering value of the recorded decisions.

## Data Availability

The original contributions presented in the study are included in the article/Supplementary material, further inquiries can be directed to the corresponding author.

## Supplementary material

Supplementary Data Sheet 1 ReqTestAgent replication artifact containing evaluation instances, implementation, prompts, archived model responses, generated tests, verifier traces, mutant definitions, aggregate results, and instructions for cached analysis and saved-test replay.

## References

[B1] Bai X. Huang S. Wei C. Wang R. (2025). Collaboration between intelligent agents and large language models: a novel approach for enhancing code generation capability. Expert Syst. Appl. 269:126357. doi: 10.1016/j.eswa.2024.126357

[B2] Barr E. T. Harman M. McMinn P. Shahbaz M. Yoo S. (2015). The oracle problem in software testing: a survey. IEEE Trans. Softw. Eng. 41, 507–525. doi: 10.1109/TSE.2014.2372785

[B3] Chantree F. Nuseibeh B. de Roeck A. Willis A. (2006). “Identifying nocuous ambiguities in natural language requirements,” in 14th IEEE international requirements engineering conference (RE'06) (Minneapolis, MN: IEEE), 59–68. doi: 10.1109/RE.2006.31

[B4] Dakhel A. M. Nikanjam A. Majdinasab V. Khomh F. Desmarais M. C. (2024). Effective test generation using pre-trained large language models and mutation testing. Inf. Softw. Technol. 171:107468. doi: 10.1016/j.infsof.2024.107468

[B5] Fan A. Gokkaya B. Harman M. Lyubarskiy M. Sengupta S. Yoo S. . (2023). “Large language models for software engineering: survey and open problems,” in 2023 IEEE/ACM international conference on software engineering: future of software engineering (ICSE-FoSE) (Melbourne, VIC: IEEE), 31–53. doi: 10.1109/ICSE-FoSE59343.2023.00008

[B6] Fan W. Ding Y. Ning L. Wang S. Li H. Yin D. . (2024). “A survey on RAG meeting LLMs: towards retrieval-augmented large language models,” in Proceedings of the 30th ACM SIGKDD conference on knowledge discovery and data mining (Barcelona: ACM), 6491–6501. doi: 10.1145/3637528.3671470

[B7] Femmer H. Méndez Fernández D. Wagner S. Eder S. (2017). Rapid quality assurance with requirements smells. J. Syst. Softw. 123, 190–213. doi: 10.1016/j.jss.2016.02.047

[B8] Ferrari A. Gori G. Rosadini B. Trotta I. Bacherini S. Fantechi A. . (2018). Detecting requirements defects with NLP patterns: an industrial experience in the railway domain. Empir. Softw. Eng. 23, 3684–3733. doi: 10.1007/s10664-018-9596-7

[B9] Fischbach J. Frattini J. Vogelsang A. Mendez D. Unterkalmsteiner M. Wehrle A. . (2023). Automatic creation of acceptance tests by extracting conditionals from requirements: NLP approach and case study. J. Syst. Softw. 197:111549. doi: 10.1016/j.jss.2022.111549

[B10] Inozemtseva L. Holmes R. (2014). “Coverage is not strongly correlated with test suite effectiveness,” in Proceedings of the 36th international conference on software engineering (Hyderabad: ACM), 435–445. doi: 10.1145/2568225.2568271

[B11] Jia Y. Harman M. (2011). An analysis and survey of the development of mutation testing. IEEE Trans. Softw. Eng. 37, 649–678. doi: 10.1109/TSE.2010.62

[B12] Just R. Jalali D. Inozemtseva L. Ernst M. D. Holmes R. Fraser G. (2014). “Are mutants a valid substitute for real faults in software testing?,” in Proceedings of the 22nd ACM SIGSOFT international symposium on foundations of software engineering (Hong Kong: ACM), 654–665. doi: 10.1145/2635868.2635929

[B13] Lemieux C. Inala J. P. Lahiri S. K. Sen S. (2023). “CodaMosa: escaping coverage plateaus in test generation with pre-trained large language models,” in 2023 IEEE/ACM 45th international conference on software engineering (ICSE) (Melbourne, VIC: IEEE), 919–931. doi: 10.1109/ICSE48619.2023.00085

[B14] Lewis P. Perez E. Piktus A. Petroni F. Karpukhin V. Goyal N. . (2020). “Retrieval-augmented generation for knowledge-intensive NLP tasks,” in Advances in Neural Information Processing Systems, Vol. 33, eds. Larochelle H. Ranzato M. Hadsell R. Balcan M. F. and Lin H. (Curran Associates, Inc.), 9459–9474. Available online at: https://proceedings.neurips.cc/paper/2020/hash/6b493230205f780e1bc26945df7481e5-Abstract.html (Accessed October 8, 2026).

[B15] Luitel D. Hassani S. Sabetzadeh M. (2024). Improving requirements completeness: automated assistance through large language models. Requir. Eng. 29, 73–95. doi: 10.1007/s00766-024-00416-3

[B16] Madaan A. Tandon N. Gupta P. Hallinan S. Gao L. Wiegreffe S. . (2023). “Self-refine: iterative refinement with self-feedback," in Advances in Neural Information Processing Systems, Vol. 36, eds. Oh A. Naumann T. Globerson A. Saenko K. Hardt M. and Levine S. (New Orleans, LA: Curran Associates, Inc.), 46534–46594. doi: 10.52202/075280-2019

[B17] Papadakis M. Kintis M. Zhang J. Jia Y. Le Traon Y. Harman M. (2019). “Mutation testing advances: an analysis and survey,” in Advances in computers, Vol. 112, ed. Memon A. M. (Amsterdam: Elsevier), 275–378. doi: 10.1016/bs.adcom.2018.03.015

[B18] Schäfer M. Nadi S. Eghbali A. Tip F. (2024). An empirical evaluation of using large language models for automated unit test generation. IEEE Trans. Softw. Eng. 50, 85–105. doi: 10.1109/TSE.2023.3334955

[B19] Schick T. Dwivedi-Yu J. Dessi R. Raileanu R. Lomeli M. Hambro E. . (2023). “Toolformer: language models can teach themselves to use tools,” in Advances in Neural Information Processing Systems, Vol. 36, eds. Oh A. Naumann T. Globerson A. Saenko K. Hardt M. and Levine S. (New Orleans, LA: Curran Associates, Inc.), 68539–68551. doi: 10.52202/075280-2997

[B20] Shinn N. Cassano F. Gopinath A. Narasimhan K. Yao S. (2023). “Reflexion: language agents with verbal reinforcement learning,” in Advances in Neural Information Processing Systems, Vol. 36, eds. Oh A. Naumann T. Globerson A. Saenko K. Hardt M. and Levine S. (New Orleans, LA: Curran Associates, Inc.), 8634–8652. doi: 10.52202/075280-0377

[B21] Wang C. Pastore F. Goknil A. Briand L. Iqbal Z. (2015). “Automatic generation of system test cases from use case specifications,” in Proceedings of the 2015 international symposium on software testing and analysis (Baltimore, MD: ACM), 385–396. doi: 10.1145/2771783.2771812

[B22] Wang C. Pastore F. Goknil A. Briand L. C. (2022). Automatic generation of acceptance test cases from use case specifications: an NLP-based approach. IEEE Trans. Softw. Eng. 48, 585–616. doi: 10.1109/TSE.2020.2998503

[B23] Wang J. Huang Y. Chen C. Liu Z. Wang S. Wang Q. (2024). Software testing with large language models: survey, landscape, and vision. IEEE Trans. Softw. Eng. 50, 911–936. doi: 10.1109/TSE.2024.3368208

[B24] Wang Z. Guo X. Tsuchiya T. (2025). “Retrieval-augmented generation for software requirement-based test case generation,” in 2025 25th international conference on software quality, reliability and security (QRS) (Hangzhou: IEEE), 108–119. doi: 10.1109/QRS65678.2025.00022

[B25] Yang D. Xie X. Yang X. Hu M. Huang Y. Zhang Y. . (2026). Assessing the impact of requirement ambiguity on LLM-based function-level code generation. *arXiv* [Preprint]. *arXiv:2604.21505*. doi: 10.48550/arXiv.2604.21505

[B26] Yang J. Jimenez C. Wettig A. Lieret K. Yao S. Narasimhan K. . (2024a). “SWE-agent: agent-computer interfaces enable automated software engineering,” in Advances in Neural Information Processing Systems, Vol. 37, eds. Globerson A. Mackey L. Belgrave D. Fan A. Paquet U. Tomczak J. and Zhang C. (Vancouver, BC: Curran Associates, Inc.), 50528–50652. doi: 10.52202/079017-1601

[B27] Yang L. Yang C. Gao S. Wang W. Wang B. Zhu Q. . (2024b). “On the evaluation of large language models in unit test generation,” in Proceedings of the 39th IEEE/ACM international conference on automated software engineering (Sacramento, CA: ACM), 1607–1619. doi: 10.1145/3691620.3695529

[B28] Yao S. Zhao J. Yu D. Du N. Shafran I. Narasimhan K. R. . (2023). “ReAct: synergizing reasoning and acting in language models,” in International conference on learning representations (Kigali). Available online at: https://openreview.net/forum?id=WE_vluYUL-X (Accessed October 8, 2026).

[B29] Yuan Z. Liu M. Ding S. Wang K. Chen Y. Peng X. . (2024). Evaluating and improving ChatGPT for unit test generation. Proc. ACM Softw. Eng. 1, 1703–1726. doi: 10.1145/3660783

[B30] Zakeri-Nasrabadi M. Parsa S. (2024). Natural language requirements testability measurement based on requirement smells. Neural Comput. Appl. 36, 13051–13085. doi: 10.1007/s00521-024-09730-x

[B31] Zhao L. Alhoshan W. Ferrari A. Letsholo K. J. Ajagbe M. A. Chioasca E.-V. . (2021). Natural language processing for requirements engineering: a systematic mapping study. ACM Comput. Surv. 54, 1–41. doi: 10.1145/3444689

[B32] Zhuo T. Y. Vu M. C. Chim J. Hu H. Yu W. Widyasari R. . (2025). “BigCodeBench: benchmarking code generation with diverse function calls and complex instructions,” in International conference on learning representations, eds. Yue Y. Garg A. Peng N. Sha F. and Yu R. (Singapore) 66602–66656. Available online at: https://proceedings.iclr.cc/paper_files/paper/2025/hash/a6a90bcc2aa470c3871b2d39a67d26e8-Abstract-Conference.html (Accessed October 8, 2026).

